# Long-Term Care and the State-Family Nexus in Italy and Japan—The Welfare State, Care Policy and Family Caregivers

**DOI:** 10.3390/ijerph20032027

**Published:** 2023-01-22

**Authors:** Rie Miyazaki

**Affiliations:** Department of Economics, Ohtsuki City College, Ohtsuki City, Yamanashi 401-0012, Japan; riemiyazakik@gamil.com or miyazaki@ohtsuki.ac.jp

**Keywords:** family caregivers, long-term care, familialism, work–care balance, Italy, Japan

## Abstract

This study aims to identify the state–family nexus in long-term care (LTC) provision for older adults in Italy and Japan which have been considered to be a familialistic welfare state and the most ageing societies in the world. Based on the more developed theoretical approach of the familialism–defamilialization continuum of care, represented by Saraceno (2016), the public policy systems as well as the LTC provision and the work–LTC reconciliation of family caregivers in particular, were compared between Italy and Japan. While both countries have lower level of institutional care, and particularly high proportions of family caregivers with relatively heavy care burdens, the share of cash-based and home care as well as the age range and family relationship of family caregivers significantly differ. Focusing on the peculiarities of LTC that the state–(market)–family cannot always be clearly separated, this study identified that the size of public expenditure, i.e., the role of the state does not immediately lead to a defamililization of care. This can contribute to the policy making for care provision and work–LTC reconciliation in several countries that will become super-aging societies in the coming decades.

## 1. Introduction

Care has long been recognized as “one of the key activities connecting state and society” [1] (p. 251). In addition, work–family, more specifically, work–care reconciliation policies have long been key issues in post-industrial societies [2,3]. However, especially after the Great Recession between the late 2000s and early 2010s, care policies in highly ageing countries have tended to shift from a social insurance-based “contribution in the past” to a social investment-based “contribution in the future” system, also due to the increasing budgetary pressures [4]. Compared to child-care policy, which is for the future working generation and promotes women/mothers’ participation in the labor market, long-term care (LTC) policy for older adults has been more neglected and given less consideration to family caregivers and their work–care life balance.

However, in most economically developed countries, both rapid population ageing, and (much) lower female employment participation than men’s remain or will remain important challenges to be faced, and much of the care for older adults is still provided by family members, especially by unemployed and part-time employed women. In this perspective, LTC policy for older adults should also be an investment-based “contribution in the future” system.

For LTC policy for older adults to become more of an investment-based “contribution in the future” system, it is necessary to analyze in more detail not only the role of the state and the family, which are the most important LTC providers (whether in cash or in kind) but also their linkages. In contrast, in the most previous studies, the role of the “state” on LTC, were analyzed, such as by the size of public LTC expenditure relative to GDP, or percentage and number of public LTC workers in the total workforce, which often hindered a proper understanding of the state–family nexus in LTC sector.

In 2019, Japan and Italy recorded the first- and second-highest ratio of the population aged 65 and over in the world, respectively (Jp: 28%; It: 23%) [5]. Japan and Italy have been frequently classified as familialistic welfare states with “strong male breadwinner” models [6,7] in which family, practically women in families with less labor market participation, have more intensified care responsibility due to the strong gender division of labor [8,9]. Considering these common demographic and welfare state characteristics, both countries are among the first to face the most critical situations regarding the LTC supply and work–LTC reconciliation of family caregivers.

The aim of this paper is to identify the state–family nexus in long-term care (LTC) provision for older adults in Italy and Japan. For this aim, after discussing the conceptual framework, in the next section, the following four research questions from a comparison between Italy and Japan will be addressed and discussed:

1. What are the characteristics of the welfare state configuration and LTC policy development?
2. In what form of benefit does the state provide LTC to older adults and what is the level of access to public LTC for them?
3. Who is the main caregiver in the family?
4. Given the first and second points, what are the characteristics of the work–family reconciliation of family caregivers?

In the sections that follow, we discuss the conceptual framework with a focus on the complexities of state and family nexus in the LTC provision for older adults. Then, for the first research question above, the welfare state configuration, LTC policy development, the LTC spending, workers and recipients will be discussed. For the second question, the gender, age and family relationships of primary family caregivers will be analyzed. Then, the work–LTC reconciliation of family caregivers will be discussed for the third question, before concluding with a summary of our findings.

## 2. Conceptual Framework—Revisiting the Role of the State and Family in the LTC Provision

Developed from the discussions of welfare states and regimes since the 1990s, the variations in care policies and those in the care models and state–market–family nexus in major European countries have been examined [10,11]. Additionally, since the 2010s, due to the Great Recession and austerity programs, there has been a growing interest in re-familialization [4,5], as well as the marketization, globalization and deskillization of social care [12]. Generally, these studies have revealed the varieties of nation–market–family nexus in a macro-perspective, and the role of the state in the state–market–family nexus has been examined and determined by the size of public LTC expenditure relative to GDP, or share or numbers of public LTC workers in the total workforce. What we should pay attention to here is that even if the spending on public care as a percentage of GDP is larger, it does not directly mean a smaller role of the family and/or market and vice versa. A good example is cash-for-care and the day care system in the LTC sector.

Cash (and/or voucher)-for-care system is one of the main state-based or public-based forms of care provision in major European countries, especially in Italy with “Accompanying Allowance (Indennità di accompagnamento)” [13,14,15]. However, in practice, this cash-for-care provided by the “state” transfers from the “state” to “market” as well as to the “family”, depending on the design of the system in each country: While the cash-for-care can be converted to a wage for formal care workers where the use of cash is controlled and restricted, it can also be a “routed wage” for unskilled workers in the “market” or, frequently, for undeclared workers including undocumented migrants who do not have citizenship in the “grey market” [16]. In other cases, it is ambiguously absorbed in the “family”, as household income of unpaid family caregivers [15].

On the other hand, a public day care system that provides care services for people in need of temporary care in day care centers is part of the “state”, however, day care services have also contributed greatly to relieving the physical and mental burden of family caregivers of people with severe care needs, such as those with dementia patients, and are an important form of respite care [17]. Thus, the day care provided by the “state” would largely depend on the care provision by “family”, especially when the public day care (and institutional care) provision is modest.

In both cases of cash-for-care and day care systems, LTC provision by the “state” is not possible without that of “family” (or “market”), and these two cannot be seen as completely independent and separate. Furthermore, in the situations above discussed, an increase and more widespread of the LTC provided by “state” may increase care provided by “family”, and lead to the familialization or re-familialization of LTC rather than the defamilialization as has been generally considered to be the case.

The best examples are the Italian and Japanese cases. As an Italian care (supply) model, the migrant-in-the-family model, which mainly consists of immigrant domestic workers directly hired by individual households [18] has been well known. This care model, the “familialization-defamiliarization continuum”, has been further elaborated and distinguished by Saraceno (2016), having a more detailed care configuration divided into five (or six) patterns by particularly looking at policy impacts on the family and the market.

As for Japan, relatively contrasting LTC provision models have been identified. On the one hand, it was considered that the Japan’s state–market–family–community nexus in the LTC supply have changed since 2000, when the National Long-Term Care Insurance (NLTCI) system has enforced and enabled public coverage of market and quasi-market care services [19,20], and role of the ‘state’ and the ‘market’ contained within the state has greatly expanded. On the other hand, the role and weight of the ‘family’ in LTC provision has not changed significantly and remains large even after 2000, and Japan has more a state-family based familialistic model [21,22]. Indeed, the LTC supply in the NLTCI is still based on the assumption that family members provide 75% of the home care required for older adults [23], and the role of the ‘market’ in Japan’s LTC supply for older adults is seen as the lowest among the other East Asian countries [24].

Among East Asian and southern European countries, the need for comparative care study has long been pointed out [2,8,25,26,27]. The previous comparative LTC studies including the cases of Italy and Japan were very influential, important and relatively new: Estevez-Abe and Naldini [20] from a political and institutional perspective on care policy making and reform, Saraceno [14] from a further developed concept of the familialism–defamilialization continuum, and Theobald et al. [12] from marketization and market-oriented reforms into home-based care by LTC policy, national statistics and workers analysis. However, none of these studies focused on both the state–family nexus in LTC supply and the work–LTC reconciliation of family caregivers under it, as mentioned above. Investigating both these issues contribute in the most aging countries in the world will contribute global LTC policy studies for older adults as well as the future policy discussions on new policy for work–LTC reconciliation of family caregivers.

## 3. Welfare State Configurations—The Narrowest Welfare States?

While there are huge geographical and cultural differences, interestingly, the following four common welfare system characteristics are identified between Italy and Japan, from the end of the 20th century to the present in the first half of the 21st century:

- The lowest social expenditures on the working-age population, such as income transfer, family allowances, active labor market policy and unemployment benefits [6,8,9,14,20,28];
- The highest social expenditures on old-age-related areas, such as pension systems and health care services [29,30,31];
- High levels of spending on compensatory social policies and little on investment-related social policies [32];
- The greatest gender inequalities in political participation, economic activities, and domestic and care work in the family [20,33].

In fact, apart from the commonalities discussed above, more recent data show that between Italy and Japan, the higher level of social expenditure, a very high level of government debt, public spending on old-age-related social and pension expenditures, and a much lower level of income support to the working-age population were commonly identified (Table 1).

In both countries, since 2000, the trend of defamilialization in LTC provision for older adults has been changing and gradually intensifying in contrasting ways. In Italy, in addition to the changes in the Italian care model mentioned above, the EUROCARE survey, Hank & Buber [35] and other relevant studies have identified that familialism in care supply in Italy and southern European countries is not always the strongest in Europe [36,37]. Additionally, the Italian third sector (terzo settore) in the mixed economy, which is among the most advanced in developed countries, has contributed an increasing share of social care providers [38].

In Japan, under NLTCI implemented in 2000, all citizens aged 40 and over are insured, and the premium is determined by their previous year’s income, i.e., based on the ability-to-pay principle. Prices for all services are predetermined, and to use these services, certification with the specific degree of care needs (from all 7 levels) must be acquired, and a care plan indicating the LTC services to be used must be drawn up. Additionally, the LTC recipients pay 10% of the total cost of the service they use as their individual payments. Between 2000 and 2020, the number of citizens (mostly aged 65 and over) receiving in-kind care services increased from 1.49 million to 4.94 million [39].

With the increase in users, the total cost of NLTCI almost tripled from 3.6 trillion in 2000 to 11.0 trillion in 2018, and the average premiums for those aged 65 and over more than doubled from 2911 yen in 2000 to 6014 yen in 2021 [39].

The contrasting LTC policy developments and current LTC policy in Japan and Italy after 2000 can be summarized in the following three-fold aspects:

- The Japanese national LTC system has been covered by mandatory national insurance since 2000, providing standardized in-kind services for persons in need of care [39]. In Italy, this system does not exist, and remarkable gaps among local governments were identified in the care provisions provided [40];
- In Japan, the national LTC system provides only in-kind services, not cash payments [39]. In contrast, in Italy, cash benefits are much more prevalent than in-kind benefits, and a unique national system for LTC is a cash allowance system called “Indennità di Accompagnamento (Accompanying Allowance)” which has no restrictions on its use [14,15,38];
- The Italian cash-oriented care system encourages the employment of less skilled and cheaper care workers, who are mostly migrants, in the (often grey) market [41]. In contrast, the Japanese in-kind-based public care system, which assigns only 10% of fixed care costs to service recipients, curbs the care services available for purchase on the market [39].

## 4. LTC Spending, Workers and Recipients

The variety of the welfare state configurations and LTC policies between Italy and Japan reveals different characteristic of the detailed LTC conditions, especially when compared with the EU average data. Table 2 shows the latest details of public spending for the LTC sector in 2019. Between Italy and Japan, while the share of public spending for the LTC sector to the GDP is almost identical (It: 1.7%; JP: 1.8%), the share of cash benefits as a percentage of public spending for LTC differs radically: Italy’s share is more than 25% higher than the EU27 average, accounting for more than half of all their LTC public transfers (52.3%), whereas it is absent in Japan (0%). The share of home care in total LTC expenditure is modestly lower than the EU27 average in Italy (19.5%) in contrast to Japan (44.7%) which is approximately more than 20% higher than the EU27 average and Italy. The share of institutional care between Italy (28.2%) and Japan (33.1%) is relatively restrained, and both countries have lower values than the EU27 average.

Table 3 shows the details of access to LTC. The share of older adults who receive cash benefits is 0% in Japan, compared to 10.9% in Italy (and 8.8% in the EU27). Considering the higher share of cash benefit in total LTC spending, the cash benefits are considered to be paid more intensively to a smaller number of people in Italy. The share of home care users in the population aged 65 and over in Japan (10.7%) is much higher than the EU27 average (5.8%) as well as that of Italy (4.7%), and this value in Japan is rather close to that of Scandinavian countries, such as Finland, Denmark and Sweden, where it exceeds 10%. Additionally, compared to the EU27 average, a lower proportion of the older adult population aged 65 and over receiving LTC in institutions is identified both in Japan and Italy.

The situations of LTC workers and informal caregivers are shown in Table 4. The difference between Japan and Italy in public LTC workers as a percentage of the population aged 65 and over is only 0.1%, and both countries’ values are below the EU27 average (3.8%), despite their LTC policies being consistently different. Furthermore, both in Japan and Italy, the percentage of caregivers with 20 h or more per week among all informal caregivers was overwhelmingly higher than in the EU27 average (22.2%), by more than 20 points. In contrast, the percentage of informal caregivers in the population was only 5.8% in Italy compared to 10.3% in the EU27 (no data for Japan).

## 5. Who Are the Primary Family Caregivers?

**Figure 1:**
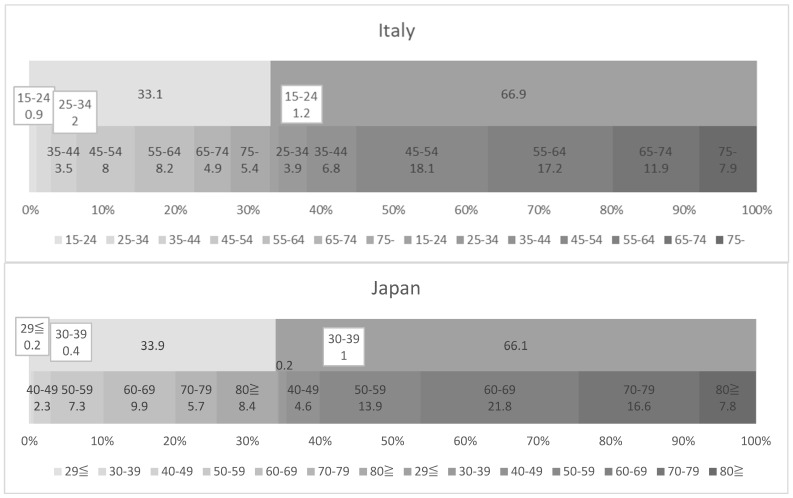
Gender and age group of the main caregivers in Italy and Japan. Source: [45,46].

Using the national survey called “Health conditions and use of health services in Italy and in the European Union (Condizioni di salute e ricorso ai servizi sanitari in Italia e nell’Unione europea)” conducted by Italian National Institute of Statistics (ISTAT) in 2016 for Italy and the Comprehensive Survey of Living Conditions conducted by the Ministry of Health, Labour and Welfare in June 2016 for Japan, the age groups and gender of major family caregivers in Italy and Japan were compared, as shown in Figure 1.

In contrast to EU member states in which comparable data on intrafamily care provisions and their sociocultural norms are covered by large-scale cross-national survey projects, such as SHARE and EUROFAMCARE, these kinds of projects and data sets are almost nonexistent between Italy and Japan. Although these limitations on comparative analysis must be taken into account, to clarify this question, this section provides a descriptive analysis by comparing the age groups of primary caregivers in Italy and Japan using their national surveys. For Italy, a national survey called “Health conditions and use of health services in Italy and in the European Union (Condizioni di salute e ricorso ai servizi sanitari in Italia e nell’Unione europea)” conducted by the Italian National Institute of Statistics (ISTAT) was used. This survey was a part of the European Health Interview Survey (EHIS), in which all EU member states participated. The EHIS was required by Commission Regulation (EU) No 141/2013 of 19 February 2013 (implementing Regulation (EC) No 1338/2008 of the European Parliament and of the Council on Community Statistics on Public Health and Health and Safety at Work) and included in the National Statistical Programme 2014–2016 (IST code 02565), with the aim of comparing the situation in different EU member states regarding the main aspects of the population’s health status and use of health services. The first edition of EHIS (that of Wave 1) was carried out between 2006 and 2009, but Italy did not participate in the survey and instead took part only in the second wave, in 2015. In Italy, the EHIS survey with the Italian title indicated above was carried out between October and December 2015. In the survey, there were 25 questionnaire items, one of which was “care or assistance provided (cure o assistenza fornite)”, which was used for this study. The survey was conducted in all regions across the country, with a sample size of 15,934 families and 29,210 individuals, and the total sample size of individuals who answered the questions on family care that was used in this study was 7293. For Japan, on the other hand, the Comprehensive Survey of Living Conditions conducted by the Ministry of Health, Labour and Welfare in June 2016 was used. This survey was launched in 1986, combining two surveys on welfare administration and national health launched in 1953 and two other surveys on national living conditions and health and hygiene launched successively in 1962 and 1963. In addition, in this survey, a large-scale survey is conducted every three years, and the survey related to long-term care that is used in this comparative study is included. Especially since the introduction of the long-term care insurance system in 2000, this large-scale survey has included more detailed surveys related to long-term care to clarify the basic conditions of family caregivers and older family members they care for. The survey was conducted in all prefectures across the country except for one prefecture where a Designated Disaster of Extreme Severity occurred in 2016, with a sample size of 224,208 for the questionnaire on households and health, and the total sample size of individuals who answered the questions on long-term care that is used in this study was 6790.

While each age group of Italy and Japan has an age difference of 5 years and the data are not completely comparable, the following points were identified:

- In both countries, the gender ratios of the primary family caregivers were equivalent, with 33% males and 66% females;
- Italy has a higher proportion of younger primary carers than Japan: In Italy, 6.2% of all primary family caregivers were under the age of 35, of which 1.1% were men and 5.1% were women. In Japan, those under the age of 40 remained 1.8%, of whom 0.6% were men and 1.2% were women. When looking at the under-45 age group in Italy and the under-50 age group in Japan, there were 6.4% men and 11.9% women, for a total of 18.3% of all family primary caregivers in Italy, compared with 2.9% men and 5.8% women, for a total of 8.7%, in Japan;
- Japan has a higher proportion of much older primary carers than Italy: In Italy, primary caregivers aged 75 and over accounted for 13.3% of all caregivers, of whom 5.4% were men and 7.9% were women. In Japan, on the other hand, primary caregivers aged 80 and over accounted for 16.2% of all primary caregivers, of whom 8.4% were men and 7.8% were women;
- The gender and age group with the highest proportion among all primary caregivers was 18.1% of women aged 45–54 in Italy, followed by 17.2% of women aged 55–64, with these two groups accounting for 35.3% of the total. In Japan, on the other hand, women aged 60–69 accounted for 21.8% of the total, followed by women aged 70–79 with 16.6%, with these two groups making up 38.4% of the total.

In the surveys presented above, the family relationship between primary caregivers and persons in need of care was identified for Japan but not for Italy which can be inferred to some extent from the work of Quattrini et al. [37] and Di Rosa et al. [47]. Although the limitations of these data should be considered, the main characteristics of the family relationships of the primary family caregivers in both countries can be summarized as follows:

- Spouses account for the highest share of all primary family caregivers, at 42.9%, in Japan. In Italy, on the other hand, spouses accounted for the third highest percentage, at 10.9%;
- Children represented 37.1% of coresident family primary caregivers in Japan, the second-largest group. In contrast, children constituted 60.9% of caregivers in Italy, making them the largest group;
- Daughters and/or sons in law represented the third-largest group of caregivers in Japan (16.5%) and the fourth largest in Italy (9.7%).

In Japan, most family caregivers were the same generation as care recipients, that is, their spouses and partners. In contrast, in Italy, the younger generation, such as the children of care recipients, plays an overwhelming role as primary family caregivers. This Italian characteristic that the younger generation of LTC recipients is the largest group of primary caregivers was identified not only in comparison with Japan but also in comparison with other European countries, such as Greece, the United Kingdom, Sweden, Poland and Germany, in the six-country European comparative study [47].

## 6. Work–LTC Reconciliation and Family Primary Caregivers

The work–LTC reconciliation of family caregivers is expected to be more difficult when their time for caregiving and work are longer. Furthermore, depending on the policy designs, care-giving duties of family caregivers frequently restrict their labor market participation, especially for women, and part-time work is among the most predominant ways for them to reconcile work with caregiving [48]. Particularly in lower-income households, their care duties reduce the hours of paid work [49]. Indeed, a correlation between lower labor market participation and higher care responsibility was identified in Italy [50].

Considering the above discussions, the work–LTC reconciliation will be more difficult in Italy where there are many more working-age primary caregivers than in Japan. However, from another perspective, the lower labor market participation of the working generation in Italy should not be ignored. In fact, according to the past EUROFAMCARE survey, only 29.1% of family caregivers of the disabled older adults in Italy were reported as “working”, and 83.1% of male family caregivers were “unemployed and seeking work”, compared to 9.9% of female caregivers [39] (p. 66, p. 75).

Table 5 shows the current labor market situation in Japan and Italy in 2019. Compared to Japan, the employment rate in Italy is 16.1% lower for women, and 20.8% lower for men, and it is approximately 10% lower for both men and women than the OECD average. In addition, the unemployment rate in Italy is 6.7% higher for men and 9.0% higher for women in comparison to the OECD average, while that in Japan remains in the 2% range both for men and women. Additionally, Italy’s long-term unemployment rates for both men and women are 30 percentage points higher than the OECD average, while the long-term unemployment rate for men in Japan is also relatively high (41.5%).

In sum, Italy has a much higher proportion of the working age population who are not working. Additionally, the interdependence or mutual aid relationships are more likely to be established between non-working active-age children who lack income and other means of subsistence and older frail parents who have pension income, their own home and assets but need daily care and supports in their daily lives. In Japan, in contrast, the higher labor market participation of both men and women, the higher part-time employment rate, the public LTC services provided by the NLTCI system, and the higher share of all the main caregivers who are almost of retirement age (aged 60 and over) would make LTC arrangements for family caregivers easier and conflicts in work–LTC reconciliation less likely to arise.

## 7. Results

Aiming to identity the state–family nexus in LTC provision for older adults, this study examined the welfare state configuration, LTC policies and intrafamily involvement in care for older adults in Japan and Italy, the most ageing societies. Although this study was of a preliminary and exploratory nature due to the limitations on the strictly comparable data, the key findings for the four research questions presented in the Introduction are summarized as follows:

1. Between Italy and Japan, there exists a similar welfare state configuration, such as the most “old-age-biased” redistribution, lower social spending on the working-age population, and the highest government debt among other OECD countries. Additionally, since the 2000s, both Italy and Japan represent a movement away from the former strong “familialism by default” and or “unsupported familialism” care model, which was common in both countries until the 1990s;
2. A contrasting LTC policy and provision, such as the cash-based public system with (grey-)market-based care provision frequently by migrant domestic workers in Italy, and a state-based in-kind care provision in Japan were observed especially since the 2000s. A more detailed comparison of the latest LTC-related data revealed that, despite this different LTC policy developments, both the total LTC expenditure as a percentage of GDP and the ratio of LTC workers to the older population were close, which were also lower than the EU27 average;
3. Regarding the types of LTC provided, the largest differences between the two countries were found for LTC cash benefit: Both its share in all LTC recipients and in all public spending for LTC were particularly high in Italy, compared to the EU27 average, as well as to Japan, where they were both 0%. In contrast, in both countries, the proportion of informal carers with longer caregiving hours (more than 20 h per week) was also outstandingly high compared to the EU27 average;
4. Intrafamily caregiving roles for the dependent older adults showed relevant differences. While the primary family caregivers’ gender proportion was exactly the same between Italy and Japan, they were consistently younger in Italy, with 44.4% of all primary family caregivers aged under 65, in contrast in Japan with 29.9% even under the age of 60. The primary family caregivers of older people tended to be children of working age in Italy and much older spouses in Japan.

## 8. Conclusions

The combinations of these four key findings above in Italy and Japan, can be considered to create a different balance between work and LTC giving among family caregivers: In Italy, the current combination of the old-age-biased welfare system, the presence of more physically and/or cognitively dependent frail older adults (parents) with a lower level of public in-kind service provision, and the higher level of caregiving by non-employed and unemployed adult children in a working age population who may be economically dependent on their parents also due to insufficient public support for the working age population, facilitate the interdependence between generations. This circumstance will more strongly oriented to the “domestication” of care as defined by Kröger [53], even though the Italian LTC model for older adults was once categorized by Saraceno & Keck [19] as “supported familialism” instead of “familialism by default”.

In contrast, in Japan, the combination of a higher labor market participation, the universal coverage of the NLTCI system, and the higher share of much older primary family caregivers aged 60 and over may have given rise to the lower share of adult children as primary caregivers for their older parents, at least compared to Italy. Furthermore, if we follow the classification by Da Roit et al., [15], Japan’s case with NLTCI and paid LTC leave appears to be a case of “defamilialization through public provision”. However, these two public systems alone, NLTCI and paid LTC leave, exactly provided by the “state”, do not enable family caregivers to combine care with full-time work, and even taking into account that the share of informal carers providing longer hours of care (more than 20 h per week) in Japan is at the same level as in Italy, which is much higher compared to other countries, the reality in Japan is closer to “Optional Familialism”.

Above all, since Japan is also characterized by a low support for the working-age population, middle-aged and older people, especially for men, who have left employment or are not working to care for their much older parents are considered a social issue. In 2017, 75,000 women and 24,000 men, a total of 99,000, left their jobs to care for their families (mostly older parents), and prominent losses in these family caregivers’ lifetime incomes were reported in Japan [54]. On the other hand, the high part-time employment rate, especially for women, suggests that women are more likely to balance care and work by reducing working hours. In fact, the number of part-time workers engaged in family care has increased significantly over the last two decades in contrast to the decrease in the three-generation households that have been considered the basis of intergenerational assistance in caregiving [55].

What this study identified is that despite the contrasts between the Japanese and Italian cases, under current situations, in both countries, the role of “state”, or the public provision in the LTC sector does not necessarily promote defamilization but may sometimes promote re-familization and/or enlarging the role of the “family”. For future research, the amount and burden of family caregivers’ LTC supply needs to be more specifically examined and identified, as well as that of public systems.

By 2050, the aging population in several major Western and Asian countries is expected to be in line with those of Japan and Italy today [5]. This means that the findings of this study would provide some significant implications for the global community. For future research, a more detailed, quantitative analysis is needed to identify the relationship between working-age children’s employment status, household composition, the caregiving role for older parents and the accessibility and availability of public LTC services.

## Figures and Tables

**Table 1:** Current demographic, economic and welfare conditions in Japan and Italy.

	Old-Age Dependency Ratio ^(1)^ (2020)	Life Expectancy (2019)	Total Net Public Social Expenditure ^(2)^ (2018)	Income Support to the Working Age ^(2)^ (2017)	Old-Age Social Spending ^(2)^ (2017)	General Government Debt ^(2)^ (2017)
Japan	52.0	M:84.4 F:87.4	21.9%	4.1%	10.88%	222.1%
Italy	39.5	M:83.6 F:85.7	27.9%	1.8%	13.35%	152.9%
OECD	--	M:81.0 F:83.6	20.1%	8.0%	9.97%	110.3%

Notes: ^(1)^ Old-age dependency ratio = Population aged 65+/20–64, the values are estimates. ^(2)^ As a percentage of GDP. Source: [5,28,31,34].

**Table 2:** Public Spending for LTC Sector in 2019.

	On LTC as % of GDP	On Cash Benefits as % of LTC Spending	On Home Care as % of LTC Spending	On Institutional Care as % of LTC Spending
Italy	1.7%	52.3%	19.5%	28.2%
Japan	1.8% ^(1)^	0%	44.7%	33.1%
EU27	1.7%	26.4%	25.5%	48.1%

Note: ^(1)^ The data is for 2017. Source: [34,42,43,44] (OECD 2019; European Commission 2021; MHLW 2020a, 2020b).

**Table 3:** Access to LTC in 2019.

	Share of Pop. 65+ Receiving LTC in Institution	Share of Pop. 65+ Receiving LTC at Home	Share of Pop. 65+ Receiving LTC Cash Benefits
Italy	3.2%	4.7%	10.9%
Japan	2.7% ^(1)^	10.7% ^(2)^	0% ^(3)^
EU27	3.6%	5.8%	8.8%

Source: [39,43]. Note: ^(1)^ Institutional care users of NLTCI/Insured person aged 65+ (≒population aged 65+). ^(2)^ Home care users of NLTCI/Insured person aged 65+ (≒population aged 65+). ^(3)^ There in no cash benefit in NLTCI and national LTC policy.

**Table 4:** LTC workers and caregivers.

	LTC Workers as % of Population Aged 65+	Share of Pop. Providing Informal Care	Share of Informal Carers Providing Care More than 20 h/w
Italy	1.7% (2021)	5.8% (2016)	40.5% (2016)
Japan	1.8% (2019)	--	43.7%
EU27	3.8% (2016)	10.3% (2016)	22.2% (2016)

Source: [39,43,44].

**Table 5:** Share of Labour force, Unemployment, Long-term Unemployment and Part-time Employment (%) in 2019 in Japan and Italy.

	Employment Rate (Aged 15–64)		Unemployment Rate (15–64)		Long-Term Unemployment Rate		Part-Time Employment Share	Women’s Share in Part-Time Employment
	Male	Female	Male	Female	Male	Female		
Japan	84.1	70.9	2.6	2.3	41.5	19.4	25.2	68.5
Italy	68.0	50.1	9.3	11.3	57.4	56.7	18.0	74.9
OECD	76.3	61.4	5.4	5.7	25.3	26.5	16.7	68.3

Source: [51,52].

## Data Availability

Not applicable.

## References

[1] Daly M. (2002). Care as a good for social policy. J. Soc. Policy.

[2] Daly M. (2012). Making policy for care, experience in Europe and its implications in Asia. Int. J. Sociol. Soc. Policy.

[3] Gregory A., Milner S., Windebank J. (2013). Work-life balance in times of economic crisis and austerity. Int. J. Sociol. Soc. Policy.

[4] León M., Pavolini E. (2014). Social Investment’ or Back to ‘Familism’: The Impact of the Economic Crisis on Family and Care Policies in Italy and Spain. South Eur. Soc. Politics.

[5] United Nations (2019). World Population Prospects 2019, Data Booklet.

[6] Osawa M. (2013). Governance of Livelihood Security.

[7] Tepe M., Vanhuysse P. (2010). Elderly bias, new social risks and social spending, change and timing in eight programmes across four worlds of welfare, 1980–2003. J. Eur. Soc. Policy.

[8] Bambra C. (2007). Defamilisation and welfare state regimes, a cluster analysis. Int. J. Soc. Welf..

[9] Uzuhashi T. (2011). Trend in International Welfare Policy and Choice of Japan.

[10] Leitner S. (2003). Varieties of Familiarism. Eur. Soc..

[11] Bettio F., Plantenga J. (2004). Comparing care regimes in Europe. Fem. Econ..

[12] Theobald H., Szebehely M., Saito Y., Ishiguro N. (2018). Marketisation policies in different contexts: Consequences for home-care workers in Germany, Japan and Sweden. Int. J. Soc. Welf..

[13] Ranci C., Pavolini E. (2013). Reforms in Long-Term Care Policies in Europe, Investigating Institutional Change and Social Impacts.

[14] Saraceno C. (2016). Varieties of familialism: Comparing four southern European and East Asian welfare regimes. J. Eur. Soc. Policy.

[15] Da Roit B., Le Bihan B. (2019). Cash for long-term care: Policy debates, visions, and designs on the move. Soc. Policy Adm..

[16] Timonen V., Convery J., Cahillt S. (2016). Care revolutions in the making? A comparison of cash-for-care programmes in four European countries. Ageing Soc..

[17] Tretteteig S., Vatne S., Rokstad A.M.M. (2017). The influence of day care centres designed for people with dementia on family caregivers—A qualitative study. BMC Geriatr..

[18] Bettio F., Simonazzi A., Villa P. (2006). Change in care regimes and female migration, the ‘care drain’ in the Mediterranean. J. Eur. Soc. Policy.

[19] Saraceno C., Keck W. (2010). Can We Identify Intergenerational Policy Regimes in Europe?. Eur. Soc..

[20] Estévez-Abe M., Naldini M. (2016). Politics of defamilialization: A comparison of Italy, Japan, Korea and Spain. J. Eur. Soc. Policy.

[21] Ochiai E., Abe A., Uzuhashi T., Tamiya Y., Shikata M. (2010). Reconfiguration of Care Diamond in Japan: The Impact of the LTCI on Familialism. Rev. Comp. Soc. Secur. Res..

[22] Soma N., Yamashita J. (2011). Child care and elder care regimes in Japan. J. Comp. Soc. Welf..

[23] Iwama O. (2009). The position of informal carers in policy documents and public support. Fam. Relat..

[24] Hiraoka K., Suda Y., Hiraoka K., Morikawa M. (2018). Introduction and Reform of National Long-Term Care Insurance System and Japan’s Care Regime for Older Adults. Elderly Care in East Asia: State, Region and Family.

[25] Yeandle S., Kröger T., Cass B. (2012). Voice and choice for users and carers? Developments in patterns of care for older people in Australia, England and Finland. J. Eur. Soc. Policy.

[26] Ferrera M. (2016). Resemblances that matter: Lessons from the comparison between Southern Europe and East Asia. J. Eur. Soc. Policy.

[27] Peng I. (2018). Shaping and Reshaping Care and Migration in East and Southeast Asia. Crit. Sociol..

[28] OECD (2019). Society at a Glance 2019.

[29] Lynch J. (2006). Age in the Welfare State: The Origins of Social Spending on Pensioners, Workers, and Children.

[30] Scruggs L., Allan J. (2006). Welfare-State Decommodification in 18 Oecd Countries: A Replication and Revision. J. Eur. Soc. Policy.

[31] OECD (2019). Government at a Glance 2019.

[32] Nikolai R., Morel N., Palier B., Palme J. (2012). Towards social investment? Patterns of public policy in the OECD world. Towards a Social Investment Welfare State.

[33] Budlender D. (2010). Time Use Studies and Unpaid Care Work.

[34] OECD (2019). Health at a Glance 2019.

[35] Hank K., Buber I. (2008). Grandparents Caring for their Grandchildren, Findings from the 2004 Survey of Health, Ageing, and Retirement in Europe. J. Fam. Issues.

[36] Eurobarometer (2007). Health and Long-Term Care in the European Union.

[37] Quattrini S., Melchiorre M.G., Balducci C., Spazzafumo L., Lamura G. (2006). Services for Supporting Family Carers of Older Dependent People in Europe, Characteristics, Coverage and Usage. Natl. Surv. Rep. Italy.

[38] Pavolini E., Ranci C. (2008). Restructuring the welfare state: Reforms in long-term care in Western European countries. J. Eur. Soc. Policy.

[39] (2021). MHLW, Overview of the Long-Term Care Insurance System. https://www.mhlw.go.jp/content/000801559.pdf.

[40] MLPS (Ministero del Lavoro e delle Politiche Sociali) (2010). Rapporto Sulla non Autosufficienza in Italia—2010.

[41] Simonazzi A. (2009). Care regimes and national employment models. Camb. J. Econ..

[42] European Commission (2021). Long-Term Care Report Trends, Challenges and Opportunities in an Ageing Society Country Profiles.

[43] MHLW (Ministry of Health Labour and Welfare) The Situation in Long Term Care Sector. https://www.mhlw.go.jp/content/12300000/000608284.pdf.

[44] MHLW Overview of Actual Statistics of Long-Term Care Benefit Costs in 2019. https://www.mhlw.go.jp/toukei/saikin/hw/kaigo/kyufu/19/dl/11.pdf.

[45] Istat (2017). Condizioni di Salute e Ricorso ai Servizi Sanitari in Italia e nell’Unione Europea—Indagine Ehis 2015.

[46] MHLW (2017). Comprehensive Survey of Living Conditions 2016.

[47] Di Rosa M., Merchiorre M., Lamura G. (2010). I servizi domiciliari tra reti informali ed assistenti famigliari. Psicogeriatria.

[48] Morgan K.J. (2009). Caring Time Policies in Western Europe, Trends and Implications. Comp. Eur. Politics.

[49] Mentzakis E., McNamee P., Ryan M. (2008). Who cares and how much, exploring the determinants of co-residential informal care. Rev. Econ. Househ..

[50] Pagani L., Marenzi A. (2008). The Labor Market Participation of Sandwich Generation Italian Women. J. Fam. Econ. Issues.

[51] OECD (2020). Employment Outlook 2020.

[52] OECD (2020). Labour Force Statistics 2010–2019.

[53] Kröger T. (2011). Defamilisation, dedomestication and care policy. Int. J. Sociol. Soc. Policy.

[54] Takada H. (2020). Current Status of Turnover Due to Long-Term Care.

[55] Miyazaki R. (2021). A descriptive analysis of three-generation households and mothers’ employment in Japan, 2002–2019. Int. J. Sociol. Soc. Policy.

